# Supplementary material for: Drivers and implications of alternative routes to fuels decarbonization in net-zero energy systems
Source: Nat Commun. 2024 May 10;15:3938. doi: 10.1038/s41467-024-47059-0 (PMC11087489; doi:10.1038/s41467-024-47059-0)
Supplement: Supplementary file 1 — Supplementary Information [file 41467_2024_47059_MOESM1_ESM.pdf]

Supplementary Information for

Drivers and implications of alternative routes to fuels decarbonization in net-zero energy systems

Bryan K. Mignone[a*], Leon Clarke[b,c], Jae Edmonds[d], Angelo Gurgel[e], Howard J. Herzog[e], Jeremiah X. Johnson[f], Dharik S. Mallapragada[e], Haewon McJeon[d], Jennifer Morris[e], Patrick R. O'Rourke[c,d], Sergey Paltsev[e], Steven K. Rose[g], Daniel C. Steinberg[h], Aranya Venkatesh[i§]

[a] ExxonMobil Technology and Engineering Company, Annandale, NJ, USA

[b] Bezos Earth Fund, Washington, DC, USA

[c] School of Public Policy, University of Maryland, College Park, MD, USA

[d] Pacific Northwest National Laboratory, Joint Global Change Research Institute, College Park, MD, USA

[e] Massachusetts Institute of Technology, Cambridge, MA, USA

[f] Department of Civil, Construction, and Environmental Engineering, North Carolina State University, Raleigh, NC, USA

[g] Energy Systems and Climate Analysis, EPRI, Washington, DC, USA

[h] National Renewable Energy Laboratory, Golden, CO, USA

[i] Department of Engineering and Public Policy, Carnegie Mellon University, Pittsburgh, PA, USA

* *Corresponding Author:* bryan.k.mignone@exxonmobil.com

§ *Now at* Energy Systems and Climate Analysis, EPRI, Washington, DC, USA

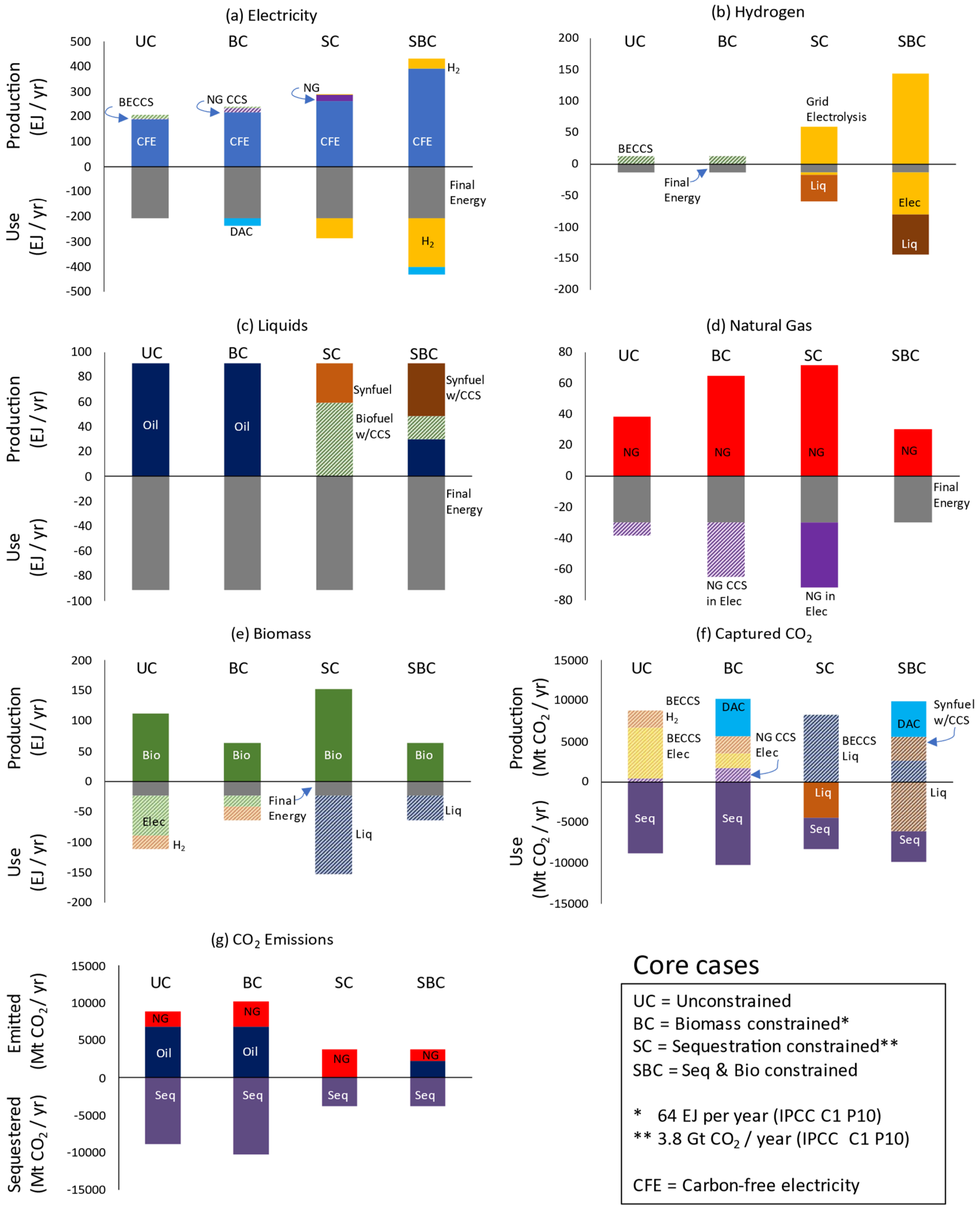

Supplementary Figure 1 | Results for the core cases. Panels (a), (b), (c) and (d) show production (by technology) and use (by sector and technology) of electricity, hydrogen, liquids, and natural gas in each of the four core cases. Panels (e) and (f) show biomass primary energy and captured CO_2 (by source), respectively, along with their final dispositions. Panel (g) shows gross positive and negative CO_2 emissions. Final energy does not vary across cases. Source data are provided as a Source Data File.

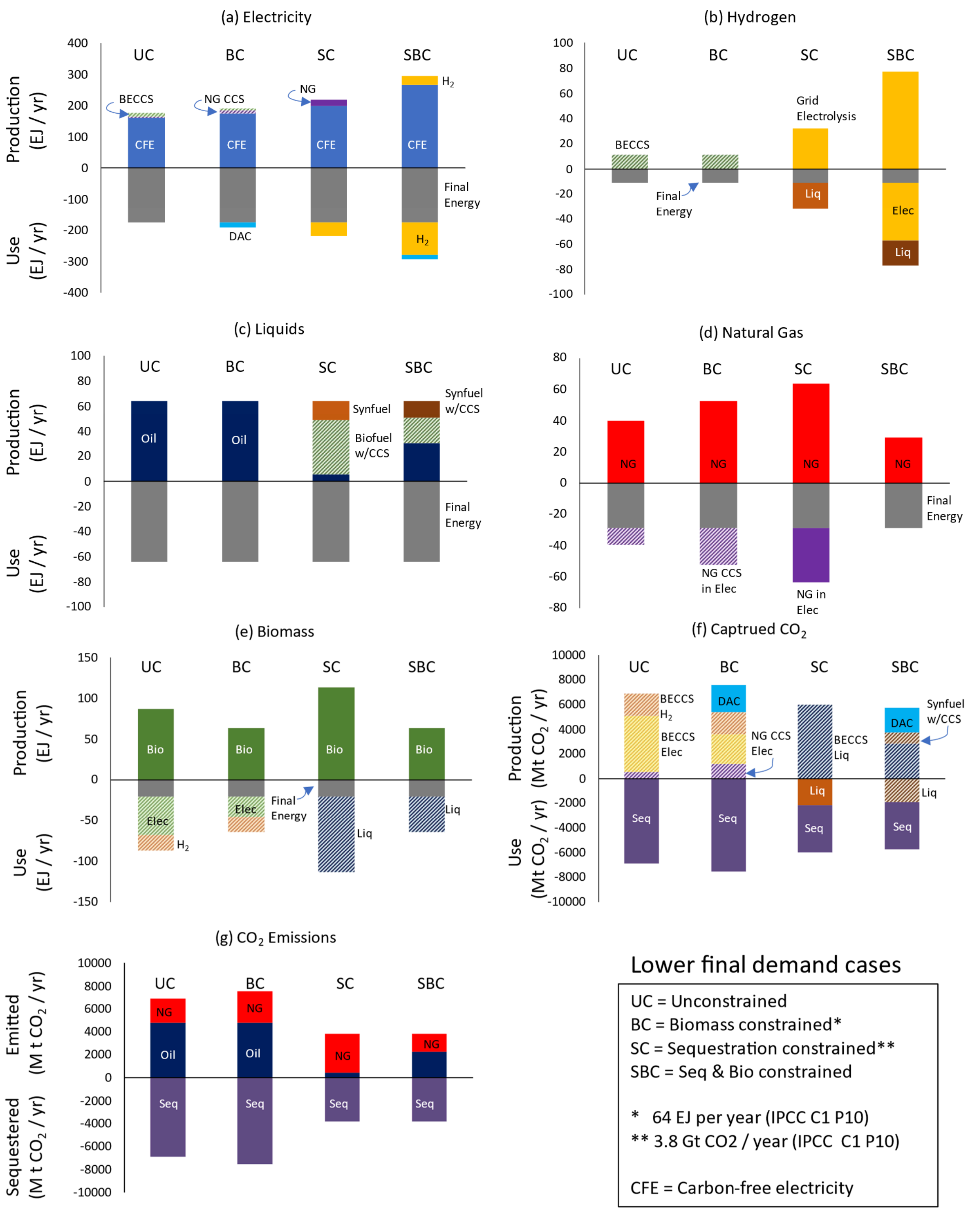

Supplementary Figure 2 | Results for lower final demand cases. Panels (a), (b), (c) and (d) show production (by technology) and use (by sector and technology) of electricity, hydrogen, liquids, and natural gas in each of the four core cases. Panels (e) and (f) show biomass primary energy and captured CO_2 (by source), respectively, along with their final dispositions. Panel (g) shows gross positive and negative CO_2 emissions. Final energy does not vary across cases. Source data are provided as a Source Data File.

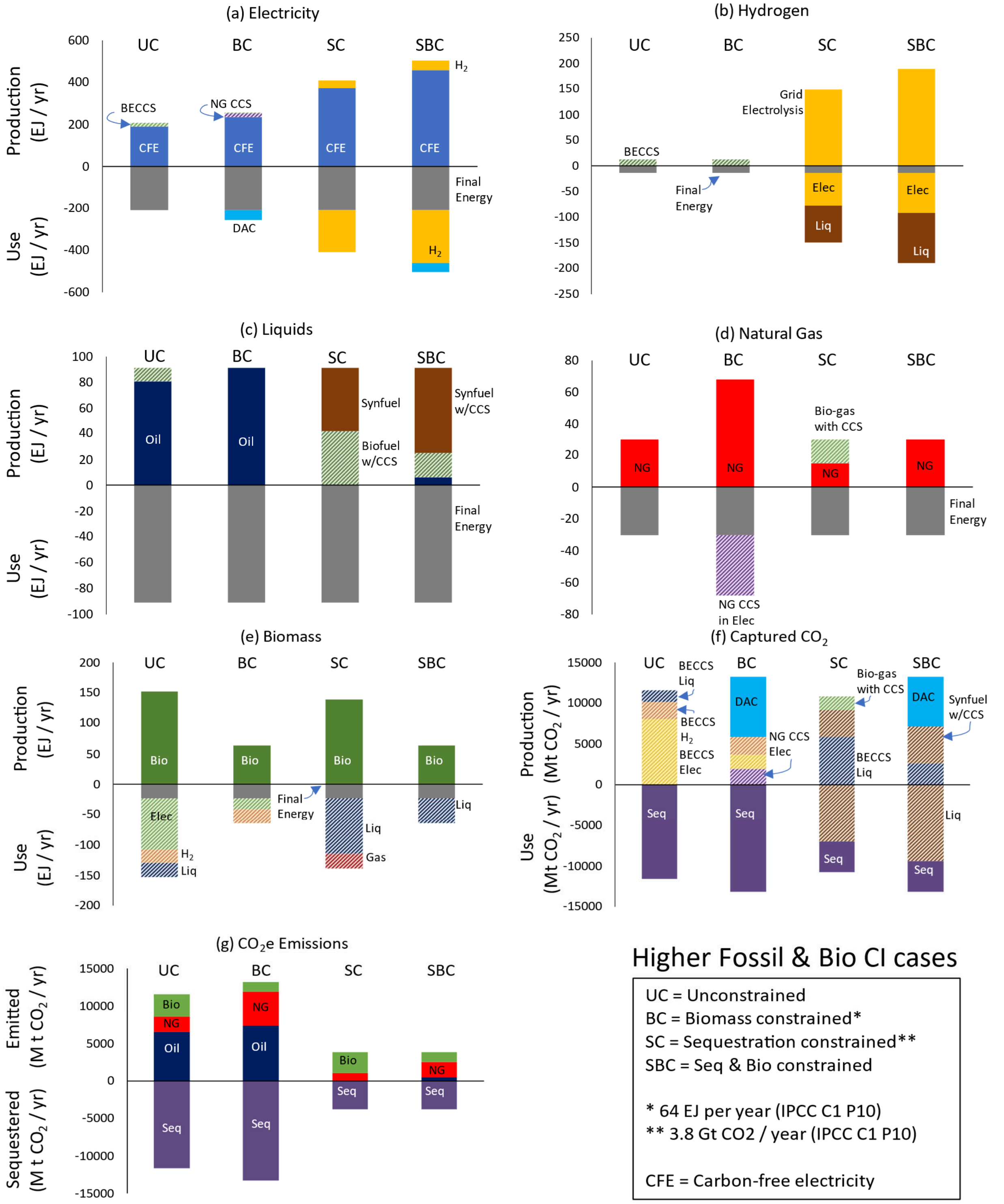

Supplementary Figure 3 | Results for cases in which the carbon intensities of oil, natural gas, and biomass are increased to reflect non-CO_2 and land-related emissions. Panels (a), (b), (c) and (d) show production (by technology) and use (by sector and technology) of electricity, hydrogen, liquids, and natural gas in each of the four core cases. Panels (e) and (f) show biomass primary energy and captured CO_2 (by source), respectively, along with their final dispositions. Panel (g) shows gross positive and negative CO_2 emissions. Final energy does not vary across cases. Source data are provided as a Source Data File.

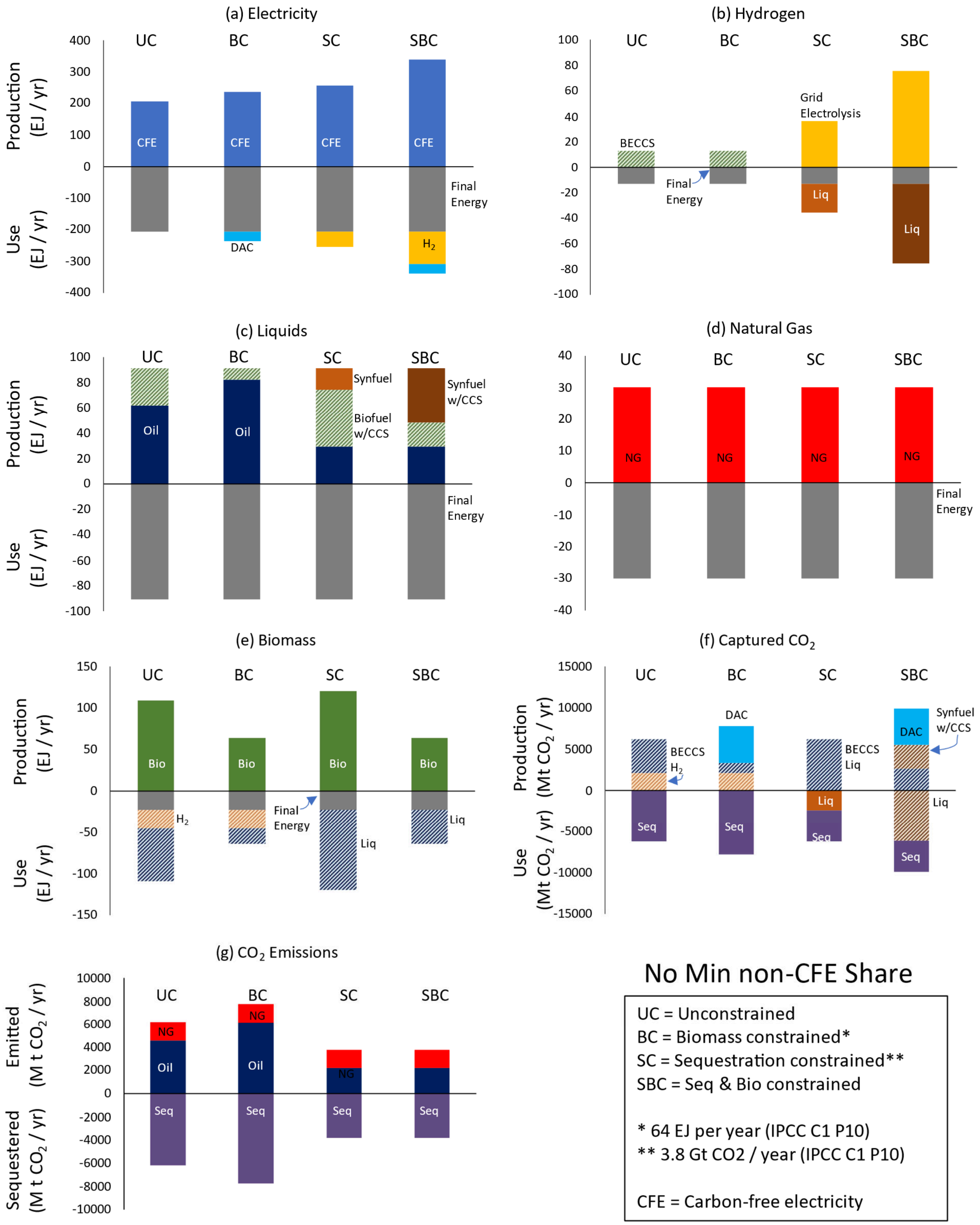

Supplementary Figure 4 | Results for cases in which the constraint on carbon-free electricity is removed. Panels (a), (b), (c) and (d) show production (by technology) and use (by sector and technology) of electricity, hydrogen, liquids, and natural gas in each of the four core cases. Panels (e) and (f) show biomass primary energy and captured CO_2 (by source), respectively, along with their final dispositions. Panel (g) shows gross positive and negative CO_2 emissions. Final energy does not vary across cases. Source data are provided as a Source Data File.

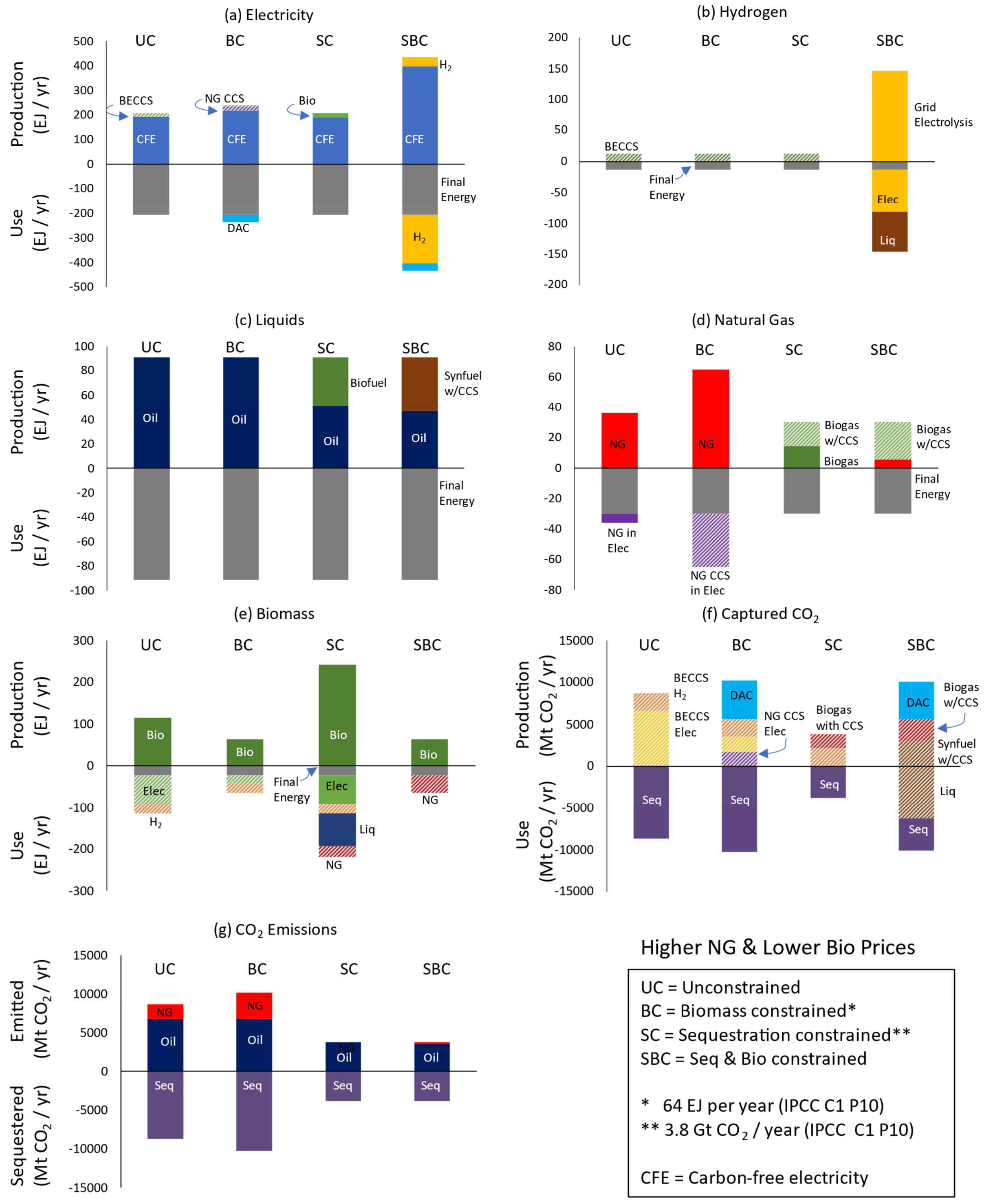

Supplementary Figure 5 | Results for cases in which NG prices are higher and biomass prices are lower than default values. Panels (a), (b), (c) and (d) show production (by technology) and use (by sector and technology) of electricity, hydrogen, liquids, and natural gas in each of the four core cases. Panels (e) and (f) show biomass primary energy and captured CO_2 (by source), respectively, along with their final dispositions. Panel (g) shows gross positive and negative CO_2 emissions. Final energy does not vary across cases. Source data are provided as a Source Data File.

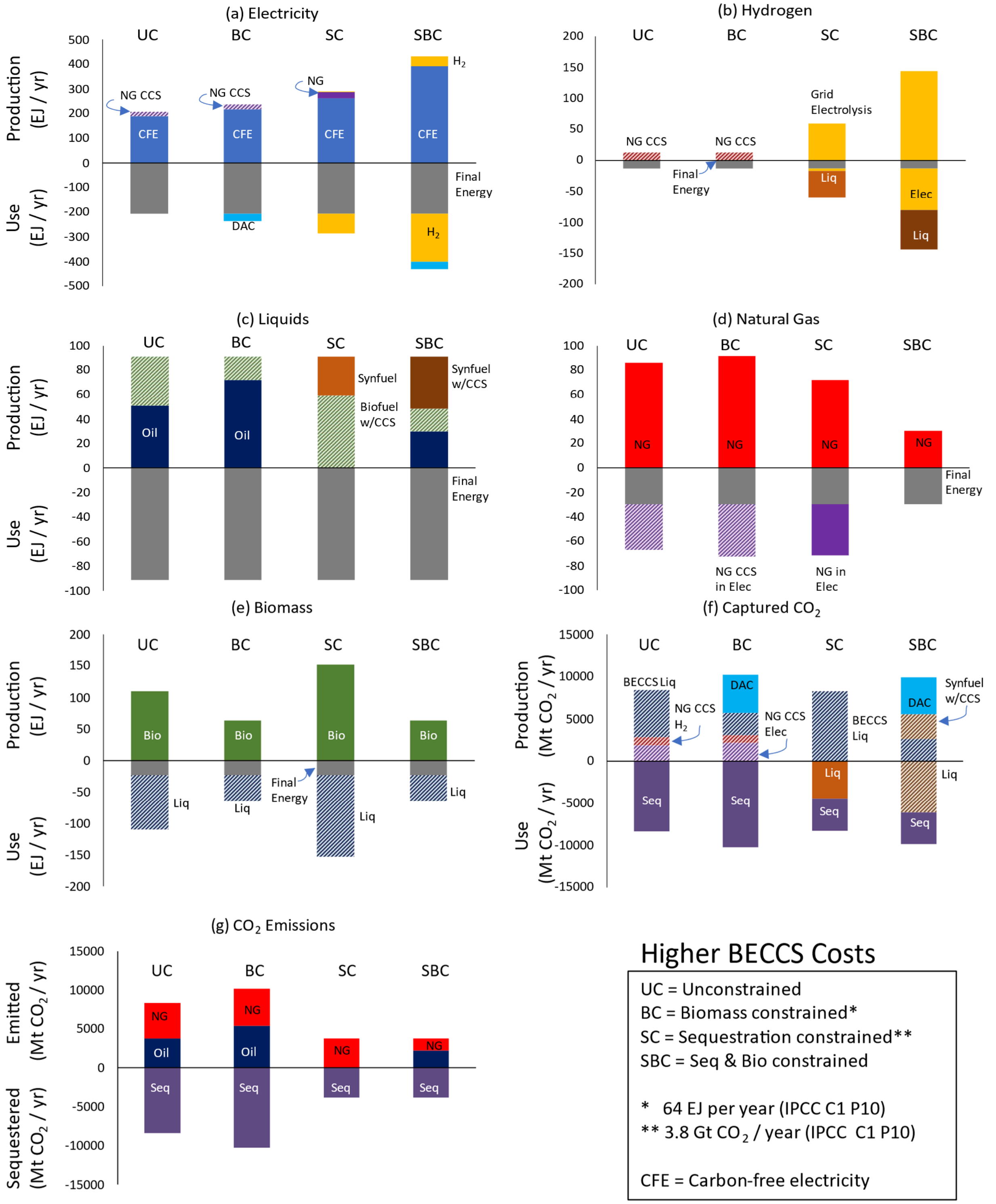

Supplementary Figure 6 | Results for cases in which BECCS electricity and BECCS H_2 non-energy costs are increased by 50% relative to default values. Panels (a), (b), (c) and (d) show production (by technology) and use (by sector and technology) of electricity, hydrogen, liquids, and natural gas in each of the four core cases. Panels (e) and (f) show biomass primary energy and captured CO_2 (by source), respectively, along with their final dispositions. Panel (g) shows gross positive and negative CO_2 emissions. Final energy does not vary across cases. Source data are provided as a Source Data File.

	IPCC AR6 C1					IEA NZ	OEO			EPRI			Williams et al		
	CDR <P10	CDR <P50	All	CDR >P50	CDR >P90		Low Bio	Det.	High Bio	Lim	All	High FC	100% RE	Cent.	Low Land
A. Net CO_2 to mitigate from fuels [B–C] (Gt)	**3.4**	**4.0**	**6.0**	**7.8**	**12.8**	**4.0**	**1.7**	**1.6**	**1.4**	**0.3**	**1.1**	**0.6**	**1.3**	**1.0**	**1.0**
B. Potential CO_2 from fuels (Gt)	7.6	7.9	8.8	9.1	10.3	4.0	1.1	1.1	1.0	0.6	1.3	0.8	0.8[§]	0.9[§]	0.9[§]
B1. Liquids [E1*CI_{oil}]	5.1	4.8	6.8	7.7	8.8	2.3	0.6	0.6	0.5	0.4	0.8	0.5	0.9	0.9	0.9
B2. Natural Gas [E2*CI_{ng}]	2.5	3.1	2.0	1.4	1.5	1.7	0.5	0.5	0.4	0.2	0.5	0.3	0.4	0.5	0.5
C. Realized Net Energy CO_2 (Gt)	4.2	3.9	2.8	1.3	-2.5	0.0	-0.6	-0.6	-0.5	0.3	0.2	0.3	-0.5	-0.1	-0.1
D. Mitigation including CDR (Gt)	**3.1**	**3.4**	**5.7**	**7.7**	**14.5**	**4.0**	**1.6**	**1.7**	**1.6**	**0.5**	**1.3**	**0.8**	**1.3**	**1.1**	**1.1**
D1. CDR: BECCS	0.5	1.9	3.7	5.8	12.1	1.3	0.1	0.5	0.5	0.0	0.8	0.4	0.0	0.3	0.6
D2. CDR: DACCS	0.0	0.0	0.3	0.0	0.0	0.6	1.1	1.0	0.6	0.0	0.0	0.1	0.0	0.0	0.1
D3. Biofuels [E1a*CI_{oil}+E2a* CI_{ng}]	2.1	1.5	1.7	1.9	2.4	1.5	0.2	0.2	0.2	0.3	0.5	0.3	0.5	0.3	0.3
D4. Synfuels [E1b*CI_{oil}+E2b* CI_{ng}]	0.5	0.0	0.0	0.0	0.0	0.6	0.2	0.1	0.3	0.3	0.0	0.0	0.8	0.4	0.1
E. Fuels (EJ)															
E1. Liquids	72	68	96	108	124	32	8.4	8.0	7.6	6.2	11.5	7.2	13.1	13.1	13.0
E1a. Bio	21	20	24	27	34	15	2.9	2.8	2.5	3.0	7.0	3.6	5.4	3.3	3.0
E1b. Synthetic	0	0	0	0	0	5	0.0	0.0	0.0	2.7	0.0	0.0	7.7	4.1	1.2
E2. Natural Gas	47	58	37	27	29	33	9.0	9.5	8.5	3.9	9.6	6.2	6.9	8.5	8.8
E2a. Bio	11	2	0	0	0	8	0.0	0.0	0.0	1.3	0.1	0.1	2.8	2.1	2.0
E2b. Synthetic	10	0	0	0	0	4	1.6	0.9	3.6	1.3	0.0	0.0	4.1	2.6	0.8

[§]Product and bunker fuel CO_2 is subtracted such that B < B1 + B2.

Supplementary Table 1 | 2050 outcomes for key variables across published studies. P50 results for Intergovernmental Panel on Climate Change (IPCC) are median outcomes from the C1 scenarios [1, 2]. The other IPCC cases are median outcomes for all C1 scenarios in which carbon dioxide removal (CDR) is less than or greater than the stated percentile. Reporting of feedstock uses of energy in the IPCC database is sparse and therefore not included here. In the International Energy Agency (IEA) net-zero case [3], ammonia and non-energy use are subtracted from IEA reported liquid fuels, and hydrogen is subtracted from IEA reported gaseous fuels. In the Open Energy Outlook (OEO) cases [4], for the purpose of this table, realized net energy CO_2 is estimated by subtracting industrial process emissions, land CO_2 and non-CO_2 emissions (estimated from known lifecycle coefficients of fuels) from net GHG emissions (assumed to be zero). The core case is the single deterministic net-zero case, and the low and high bio cases are cases with total bioenergy with carbon capture and sequestration (BECCS) deployment equal to the 10th percentile and the 90th percentile, respectively, from the larger scenario ensemble using the modeling-to-generate-alternatives (MGA) method. The three EPRI cases are the Limited Options, All Options, and Higher Fuel Cost cases from the EPRI Low-Carbon Resources Initiative (LCRI) net-zero 2050 study [5]. Three (of six) cases are shown from Williams et al [6]: the Central Case, as well as the cases with the lowest and highest amounts of CDR (100% RE and Low Land cases, respectively). Realized net energy CO_2 is estimated by subtracting industrial process emissions (known from the 100% RE case) from total energy and industrial emissions. In addition, potential CO_2 emissions from fuels is estimated by subtracting product and bunker CO_2 (reported as approximately 0.5 Gt) from the sum of B1 and B2. The allocation of fuels across different sources is estimated using the shares reported in Table 1 of Williams et al. [6]. Across all studies and cases, for ease of comparison, the carbon intensities for oil and natural gas are assumed be the same (0.071 and 0.053 ton/GJ, respectively), which may introduce small errors. Natural gas coupled with CCS is generally not included because potential emissions will be small (assuming high capture fractions), but this may introduce small errors. Errors due to rounding may also explain some differences.

	Units	2020	2050				
		P50	P10	P25	P50	P75	P90
Electricity	EJ	84	155	178	207	252	279
Liquid fuels	EJ	168	41	60	91	122	146
Natural gas	EJ	65	13	19	30	48	80
Hydrogen	EJ	0	6	7	13	19	27
Biomass	EJ	43	13	19	23	33	48
Coal	EJ	40	0	1	2	7	25
Total	EJ	414	336	370	410	448	520

Supplementary Table 2 | Median and other percentiles for final energy demand by carrier in 2020 and 2050 in 1.5°C scenarios. The 1.5°C scenarios are the set of 97 (C1) scenarios assessed by the Intergovernmental Panel on Climate Change (IPCC) that are consistent with 1.5°C stabilization with limited or no overshoot [1, 7]. Values in each row are estimated separately based on the distribution for that variable. It is worth noting that, due to the competition among these energy carriers, the set of outcomes in each column is not likely to occur within any actual scenario (e.g., scenarios with P10 outcomes for liquid fuels are likely to have electricity outcomes considerably higher than P10).

Electricity Production Technology	Capex ($ / kW)	Opex (% capex)	Efficiency
Gas	800	25%	57%
Gas CCS	1600	25%	50%
Bio	3600	25%	27%
Bio CCS (BECCS)	4800	25%	22%
CFE	600	25%	n/a
H_2	800	25%	57%

Supplementary Table 3 | Costs and conversion efficiencies for electricity technologies in the simple model. Efficiency is defined with respect to the dominant energy input (e.g., biomass for Bio, natural gas for Gas, etc.). CFE refers to carbon-free electricity (wind, solar, nuclear, and hydro). CCS refers to carbon capture and sequestration. Capital costs are based on National Renewable Energy Laboratory (NREL) Annual Technology Baseline (ATB) 2022 [8]. CFE is based on solar PV with an assumed capacity factor of 25%. Adjusting for differences in regional capacity factors, the CFE cost is comparable to the 2050 solar PV cost assumptions from the International Energy Agency (IEA) [9]. Bio CCS is based on Bio with capital costs inflated using the cost difference between coal with CCS and coal without CCS. H_2 is based on Gas with the primary difference being the cost of the fuel consumed (not shown here).

Hydrogen Production Technology	Capex ($ / kW)	Opex (% capex)	Efficiency
Gas	900	25%	76%
Gas CCS	1300	25%	69%
Bio	2700	25%	60%
Bio CCS	2800	25%	58%
Grid Electrolysis	600	25%	74%

Supplementary Table 4 | Costs and conversion efficiencies for hydrogen technologies in the simple model. Efficiency is defined with respect to the dominant energy input (e.g., biomass for Bio, natural gas for Gas, etc.). CCS refers to carbon capture and sequestration. Capital costs for Gas, Gas CCS and Grid Electrolysis are based on long-term cost estimates from the International Energy Agency (IEA) [10]. Bio and Bio CCS are based on coal gasification with and without CCS, respectively, from the same source. The resulting levelized non-energy costs for gas CCS, Bio CCS and electrolysis are comparable to the 2050 costs reported by O'Rourke et al [11], which were derived from the National Renewable Energy Laboratory (NREL) H2A [12] assumptions. The costs for Gas CCS are close to the total plant costs estimated by the National Energy Technology Laboratory (NETL) for an Nth-of-a-Kind steam methane reformer (SMR) with CCS assuming 96% capture, and the conversion efficiency is also comparable [13]. All costs are reported on an output basis ($/kW H_2).

Liquid Fuel Production Technology	Non-energy cost ($ / GJ)	Efficiency
Conventional (Oil-based)	3	95%
Bio FT	26	51%
Bio FT CCS	30	46%
Synthetic (H_2-based) FT	3	73%
Synthetic (H_2-based) FT CCS	7	68%

Supplementary Table 5 | Costs and conversion efficiencies for liquid fuel production technologies in the simple model. Efficiency is defined with respect to the dominant energy input (e.g., biomass for Bio FT, hydrogen for Synthetic FT, etc.). Non-energy cost includes capital and operating costs but not feedstock costs. CCS refers to carbon capture and sequestration. FT refers to Fischer-Tropsch processes. Assumptions for Conventional, Bio FT and Bio FT CCS follow Muratori et al. [14]. Assumptions for Synthetic FT are based on IEA [10]. Non-energy costs for Bio FT are in the range of capital plus operating expenses shown in [15] and in Kreutz et al [16]. Synthetic FT involves production of a synthetic fuel in which hydrogen and CO_2 are used as inputs to an FT synthesis process. The reported efficiency of this technology reflects the efficiency of hydrogen use. Following Zang et al. [17], it is assumed that the CO_2 utilization efficiency is near 50%, or that ~7 GJ of fuel can be produced with 1 ton CO_2. A significant share of the CO_2 that is not utilized can be captured at an additional cost (Synthetic FT CCS) and utilized again or sequestered. The cost of the Synthetic FT CCS technology is based on the Synthetic FT technology with costs inflated using the difference between Bio FT CCS and Bio FT. All costs are reported on an output basis ($/GJ liquid fuel produced).

Natural Gas Production Technology	Non-energy cost ($ / GJ)	Efficiency
Conventional (Fossil NG)	0	100%
Bio	15	65%
Bio CCS	16	60%
Synthetic (H_2-based)	3	77%

Supplementary Table 6 | Costs and conversion efficiencies for natural gas production technologies in the simple model. Efficiency is defined with respect to the dominant energy input (e.g., biomass for Bio, hydrogen for Synthetic, etc.). Non-energy cost includes capital and operating costs but not feedstock costs. For conventional (fossil) natural gas, the costs and losses associated with processing are assumed to be small relative to the cost of the fuel. CCS refers to carbon capture and sequestration. Assumptions for Bio follow [15], and assumptions for synthetic gas from methanation are based on [10]. It is assumed that the CO_2 utilization efficiency for synthetic methane is 90%. All costs are reported on an output basis ($/GJ natural gas produced).

	Units	2020	2050				
		P50	P10	P25	P50	P75	P90
CFE share		36%	67%	83%	91%	93%	95%
LE share		36%	91%	94%	95%	97%	99%
Primary bio	EJ/y	31	64	84	105	143	183
CO_2 seq	Gt CO_2/y	0	3.8	5.4	7.3	12	18

Supplementary Table 7 | Median and other percentiles for key outcomes in 2020 and 2050 in 1.5°C scenarios. The 1.5°C scenarios are the set of 97 (C1) scenarios assessed by the Intergovernmental Panel on Climate Change (IPCC) that are consistent with 1.5°C stabilization with limited or no overshoot [1, 7]. Values in each row are estimated separately based on the distribution for that variable. CFE share is the carbon-free share in electricity, where carbon-free is defined as wind, solar, nuclear, and hydro. LE share is the low-emission share, which includes the four technologies in the CFE share, as well as carbon capture and sequestration (CCS) technologies. Primary bio is the amount of modern biomass primary energy, and CO_2 seq is the amount of geologic CO_2 sequestration per year.

	Sector (EJ per year)						CO$_2$ (Mton per year)	
	Electricity	Hydrogen	Liquids	Gas	Biomass		Capture	Emissions
**Oil**	0	0	91	0	0	**oil**	0	6801
**Gas**	0	0	0	38	0	**ng**	0	2022
**Gas CCS**	4	0	0	0	0	**bio**	0	0
**Bio**	0	0	0	0	112	**e-gasccs**	411	0
**Bio CCS**	15	13	0	0	0	**e-bioccs**	6283	0
**CFE**	188	0	0	0	0	**h-gasccs**	0	0
**H2**	0	0	0	0	0	**h-bioccs**	2129	0
**FE**	-207	-13	-91	-30	-23	**l-bioccs**	0	0
**Elec**	0	0	0	-8	-66	**l-h2ccs**	0	0
**H2**	0	0	0	0	-22	**dac-elec**	0	0
**Liq**	0	0	0	0	0	**l-h2**	0	0
**Gas**	0	0	0	0	0	**l-h2ccs**	0	0
**DAC**	0	0	0	0	0	**seq**	-8823	-8823

Supplementary Table 8 | Values for the Unconstrained (UC) case in Figure 3. Positive values indicate production, whereas negative values indicate disposition, consistent with Figure 3.

	Sector (EJ per year)						CO$_2$ (Mton per year)	
	Electricity	Hydrogen	Liquids	Gas	Biomass		Capture	Emissions
**Oil**	0	0	91	0	0	**oil**	0	6801
**Gas**	0	0	0	65	0	**ng**	0	3429
**Gas CCS**	17	0	0	0	0	**bio**	0	0
**Bio**	0	0	0	0	64	**e-gasccs**	1747	0
**Bio CCS**	4	13	0	0	0	**e-bioccs**	1766	0
**CFE**	217	0	0	0	0	**h-gasccs**	0	0
**H2**	0	0	0	0	0	**h-bioccs**	2129	0
**FE**	-207	-13	-91	-30	-23	**l-bioccs**	0	0
**Elec**	0	0	0	-35	-16	**l-h2ccs**	0	0
**H2**	0	0	0	0	-22	**dac-elec**	4588	0
**Liq**	0	0	0	0	0	**l-h2**	0	0
**Gas**	0	0	0	0	0	**l-h2ccs**	0	0
**DAC**	-31	0	0	0	0	**seq**	-10230	-10230

Supplementary Table 9 | Values for the Biomass Constrained (BC) case in Figure 3. Positive values indicate production, whereas negative values indicate disposition, consistent with Figure 3.

	Sector (EJ per year)						CO_2 (Mton per year)	
	Electricity	Hydrogen	Liquids	Gas	Biomass		Capture	Emissions
**Oil**	0	0	0	0	0	**oil**	0	0
**Gas**	24	0	0	72	0	**ng**	0	3800
**Gas CCS**	0	0	0	0	0	**bio**	0	0
**Bio**	0	0	0	0	153	**e-gasccs**	0	0
**Bio CCS**	0	0	60	0	0	**e-bioccs**	0	0
**CFE**	262	60	0	0	0	**h-gasccs**	0	0
**H2**	2	0	31	0	0	**h-bioccs**	0	0
**FE**	-207	-13	-91	-30	-23	**l-bioccs**	8287	0
**Elec**	0	-4	0	-42	0	**l-h2ccs**	0	0
**H2**	-81	0	0	0	0	**dac-elec**	0	0
**Liq**	0	-43	0	0	-130	**l-h2**	-4487	0
**Gas**	0	0	0	0	0	**l-h2ccs**	0	0
**DAC**	0	0	0	0	0	**seq**	-3800	-3800

Supplementary Table 10 | Values for the Sequestration Constrained (SC) case in Figure 3. Positive values indicate production, whereas negative values indicate disposition, consistent with Figure 3.

	Sector (EJ per year)						CO_2 (Mton per year)	
	Electricity	Hydrogen	Liquids	Gas	Biomass		Capture	Emissions
**Oil**	0	0	30	0	0	**oil**	0	2210
**Gas**	0	0	0	30	0	**ng**	0	1590
**Gas CCS**	0	0	0	0	0	**bio**	0	0
**Bio**	0	0	0	0	64	**e-gasccs**	0	0
**Bio CCS**	0	0	19	0	0	**e-bioccs**	0	0
**CFE**	392	144	0	0	0	**h-gasccs**	0	0
**H2**	39	0	43	0	0	**h-bioccs**	0	0
**FE**	-207	-13	-91	-30	-23	**l-bioccs**	2623	0
**Elec**	0	0	0	0	0	**l-h2ccs**	2906	0
**H2**	-194	-68	0	0	0	**dac-elec**	4352	0
**Liq**	0	-63	0	0	-41	**l-h2**	0	0
**Gas**	0	0	0	0	0	**l-h2ccs**	-6081	0
**DAC**	-30	0	0	0	0	**seq**	-3800	-3800

Supplementary Table 11 | Values for the Sequestration and Biomass Constrained (SBC) case in Figure 3. Positive values indicate production, whereas negative values indicate disposition, consistent with Figure 3.

Constraint	Units	UC	BC	SC	SBC
Market clearing (supply ≥ demand)					
Electricity	$/GJ	14	14	16	16
Hydrogen	$/GJ	15	15	25	25
Liquid fuels	$/GJ	34	47	45	68
Natural gas	$/GJ	13	22	25	37
Biomass	$/GJ	10	27	10	37
Captured CO_2	$/ton	119	294	48	309
Imposed limits (supply ≤ X)					
Biomass	$/GJ	0	17	0	27
CO_2 Sequestration	$/ton	0	0	306	265
CFE	$/GJ	11	12	36	36
CO_2 Emissions (carbon price)	$/ton	129	304	364	584

Supplementary Table 12 | Shadow values on major constraints across the core cases.

Scenario Family	Scenario	Annual Bio limit (EJ)	Annual Seq limit (Gt CO_2)	CFE share limit	FE demand	Land CO_2 and non-CO_2 Emissions Factors	Fuel Prices (Bio and NG)	BECCS Non-energy Costs
Core (Figure 3 and Supplemental Figure 1)	UC	-	-	91%	Median C1	-	-	-
	BC	64	-	91%	Median C1	-	-	-
	SC	-	3.8	91%	Median C1	-	-	-
	SBC	64	3.8	91%	Median C1	-	-	-
Lower Final Demand (Supplemental Figure 2)	UC	-	-	91%	**Median (C1 | Tot FE < P25)**	-	-	-
	BC	64	-	91%	**Median (C1 | Tot FE < P25)**	-	-	-
	SC	-	3.8	91%	**Median (C1 | Tot FE < P25)**	-	-	-
	SBC	64	3.8	91%	**Median (C1 | Tot FE < P25)**	-	-	-
Higher Fossil & Bio CI (Supplemental Figure 3)	UC	-	-	91%	Median C1	**Included**	-	-
	BC	64	-	91%	Median C1	**Included**	-	-
	SC	-	3.8	91%	Median C1	**Included**	-	-
	SBC	64	3.8	91%	Median C1	**Included**	-	-
No Min Non-CFE Share (Supplemental Figure 4)	UC	-	-	**100%**	Median C1	-	-	-
	BC	64	-	**100%**	Median C1	-	-	-
	SC	-	3.8	**100%**	Median C1	-	-	-
	SBC	64	3.8	**100%**	Median C1	-	-	-
Higher NG Prices & Lower Biomass Prices (Supplemental Figure 5)	UC	-	-	91%	Median C1	-	**Changed**	-
	BC	64	-	91%	Median C1	-	**Changed**	-
	SC	-	3.8	91%	Median C1	-	**Changed**	-
	SBC	64	3.8	91%	Median C1	-	**Changed**	-
Higher BECCS Costs (Supplemental Figure 6)	UC	-	-	91%	Median C1	-	-	**Higher**
	BC	64	-	91%	Median C1	-	-	**Higher**
	SC	-	3.8	91%	Median C1	-	-	**Higher**
	SBC	64	3.8	91%	Median C1	-	-	**Higher**

Supplementary Table 13 | Core scenarios and sensitivity scenarios considered in this study. The final demands are 2050 values from the C1 scenarios in the Intergovernmental Panel on Climate Change (IPCC) AR6 scenarios database [1, 7]. Default assumptions are median values from the full set of C1 scenarios (Median C1), while the Lower Final Demand cases use median values from the subset of C1 scenarios in which total final demand is less than the 25th percentile (339 EJ), close to the 10th percentile of all C1 cases. Final demands for electricity, hydrogen, liquids, natural gas, and biomass in this case are 176, 11, 64, 29 and 20 EJ, respectively. The addition to the carbon intensity in the Higher Fossil & Bio CI cases are 0.006, 0.014 and 0.020 tons per GJ for oil, natural gas, and biomass, respectively, following DeCarolis et al. [18] and discussed in Methods. The default carbon-free electricity (CFE) share (91%) is discussed in Methods. The cases with alternative fuel prices use $15 per GJ for natural gas and $5 per GJ for biomass, compared with default values of $6 per GJ (natural gas) and $10 per GJ (biomass), as discussed in Methods. Cases with higher bioenergy with carbon capture and sequestration (BECCS) costs increase non-energy costs of BECCS electricity and BECCS H_2 by 50% relative to the core cases.

Scenario Family	Scenario	Primary Bioenergy (EJ / y)	CO_2 Sequestration (Gt / y)	Electricity (EJ / y)	Hydrogen (EJ / y)
Core (Figure 3 and Supplementary Figure 1)	UC	112	8.8	207	13
	BC	64	10.2	238	13
	SC	153	3.8	288	60
	SBC	64	3.8	431	144
Lower Final Demand (Supplementary Figure 2)	UC	87	6.9	176	11
	BC	64	7.6	191	11
	SC	113	3.8	219	32
	SBC	64	3.8	294	77
Higher Fossil & Bio CI (Supplementary Figure 3)	UC	152	11.6	207	13
	BC	64	13.2	257	13
	SC	139	3.8	409	150
	SBC	64	3.8	504	189
No Min Non-CFE Share (Supplementary Figure 4)	UC	109	6.2	207	13
	BC	64	7.8	237	13
	SC	120	3.8	256	36
	SBC	64	3.8	339	76
Higher NG & Lower Biomass Prices (Supplementary Figure 5)	UC	115	8.7	207	13
	BC	64	10.2	238	13
	SC	241	3.8	207	13
	SBC	64	3.8	435	146
Higher BECCS Costs (Supplementary Figure 6)	UC	110	8.4	207	13
	BC	64	10.2	238	13
	SC	153	3.8	288	60
	SBC	64	3.8	431	144

Supplementary Table 14 | Outcomes for the core scenarios and sensitivity scenarios considered in this study.

Scenario Family	Scenario	Total Liquids (EJ / y)	Fossil Liquids (EJ / y)	Total Natural Gas (EJ / y)	Fossil Natural Gas (EJ / y)
Core (Figure 3 and Supplementary Figure 1)	UC	91	91	38	38
	BC	91	91	65	65
	SC	91	0	72	72
	SBC	91	30	30	30
Lower Final Demand (Supplementary Figure 2)	UC	64	64	40	40
	BC	64	64	52	52
	SC	64	6	64	64
	SBC	64	30	29	29
Higher Fossil & Bio CI (Supplementary Figure 3)	UC	91	81	30	30
	BC	91	91	68	68
	SC	91	0	30	15
	SBC	91	6	30	30
No Min Non-CFE Share (Supplementary Figure 4)	UC	91	62	30	30
	BC	91	82	30	30
	SC	91	30	30	30
	SBC	91	30	30	30
Higher NG & Lower Biomass Prices (Supplementary Figure 5)	UC	91	91	36	36
	BC	91	91	65	65
	SC	91	51	30	0
	SBC	91	47	30	5
Higher BECCS Costs (Supplementary Figure 6)	UC	91	51	81	81
	BC	91	72	92	92
	SC	91	0	72	72
	SBC	91	30	30	30

Supplementary Table 15. Outcomes for the core scenarios and sensitivity scenarios relevant to Figure 5.

Constraint	Units	UC	BC	SC	SBC
Market clearing (supply ≥ demand)					
Electricity	$/GJ	14	14	15	16
Hydrogen	$/GJ	15	15	24	25
Liquid fuels	$/GJ	34	47	44	68
Natural gas	$/GJ	13	22	20	37
Biomass	$/GJ	10	27	10	37
Captured CO_2	$/ton	119	294	54	309
Imposed limits (supply ≤ X)					
Biomass	$/GJ	0	17	0	27
CO_2 Sequestration	$/ton	0	0	207	265
CFE	$/GJ	11	12	28	36
CO_2 Emissions (carbon price)	$/ton	129	304	271	584

Supplementary Table 16 | Shadow values on major constraints across the Lower Final Demand cases.

Constraint	Units	UC	BC	SC	SBC
Market clearing (supply ≥ demand)					
Electricity	$/GJ	14	15	16	16
Hydrogen	$/GJ	15	18	25	25
Liquid fuels	$/GJ	37	49	53	68
Natural gas	$/GJ	17	27	34	42
Biomass	$/GJ	13	29	18	37
Captured CO_2	$/ton	155	299	118	309
Imposed limits (supply ≤ X)					
Biomass	$/GJ	0	13	0	16
CO_2 Sequestration	$/ton	0	0	287	219
CFE	$/GJ	10	20	36	36
CO_2 Emissions (carbon price)	$/ton	165	309	415	538

Supplementary Table 17 | Shadow values on major constraints across the Higher Fossil and Bio CI cases.

Constraint	Units	UC	BC	SC	SBC
Market clearing (supply ≥ demand)					
Electricity	$/GJ	13	13	13	13
Hydrogen	$/GJ	14	15	21	21
Liquid fuels	$/GJ	34	46	42	59
Natural gas	$/GJ	13	22	19	31
Biomass	$/GJ	10	26	10	32
Captured CO_2	$/ton	126	287	70	287
Imposed limits (supply ≤ X)					
Biomass	$/GJ	0	16	0	22
CO_2 Sequestration	$/ton	0	0	160	177
CFE	$/GJ	0	0	0	0
CO_2 Emissions (carbon price)	$/ton	136	297	240	474

Supplementary Table 18. Shadow values on major constraints across the No Min Non-CFE Share cases.

Constraint	Units	UC	BC	SC	SBC
Market clearing (supply ≥ demand)					
Electricity	$/GJ	15	15	15	16
Hydrogen	$/GJ	19	22	23	25
Liquid fuels	$/GJ	28	48	36	68
Natural gas	$/GJ	18	32	23	46
Biomass	$/GJ	5	32	5	38
Captured CO_2	$/ton	38	305	15	309
Imposed limits (supply ≤ X)					
Biomass	$/GJ	0	27	0	33
CO_2 Sequestration	$/ton	0	0	132	265
CFE	$/GJ	23	30	28	36
CO_2 Emissions (carbon price)	$/ton	48	315	157	584

Supplementary Table 19 | Shadow values on major constraints across the Higher NG Price & Lower Biomass Price cases.

Constraint	Units	UC	BC	SC	SBC
Market clearing (supply ≥ demand)					
Electricity	$/GJ	14	14	16	16
Hydrogen	$/GJ	18	18	25	25
Liquid fuels	$/GJ	34	47	45	68
Natural gas	$/GJ	13	22	25	37
Biomass	$/GJ	10	27	10	37
Captured CO_2	$/ton	126	294	48	309
Imposed limits (supply ≤ X)					
Biomass	$/GJ	0	17	0	27
CO_2 Sequestration	$/ton	0	0	306	265
CFE	$/GJ	11	12	36	36
CO_2 Emissions (carbon price)	$/ton	136	304	364	584

Supplementary Table 20 | Shadow values on major constraints across the Higher BECCS Cost cases.

Supplementary References

[1] K. Riahi, R. Schaeffer, J. Arango, K. Calvin, C. Guivarch, H. T., K. Jiang, E. Kriegler, R. Matthews, G. Peters, A. Rao, S. Robertson, A. M. Sebbit, J. Steinberger, M. Tavoni and D. van Vuuren, "Mitigation Pathways Compatible with Long-Term Goals," in *Climate Change 2022: Mitigation of Climate Change*, J. Skea and P. R. Shukla, Eds., Intergovernmental Panel on Climate Change, 2022.

[2] E. Byers, V. Krey, E. R. K. Kriegler and R. Schaffer, "AR6 Scenarios Database hosted by IIASA," International Institute for Applied Systems Analysis, 2022.

[3] International Energy Agency, "Net Zero by 2050: A Roadmap for the Global Energy Sector," 2021.

[4] A. Sinha, A. Venkatesh, K. Jordan, C. Wade, H. Eshraghi, A. de Queiroz, P. Jaramillo and J. Johnson, "Diverse Decarbonization Pathways Under Near Cost-Optimal Futures," 2023.

[5] G. Blanford, N. Kern, S. Rose, N. Johnson, C. Roney, S. Goteti, J. Bistline, D. Young, C. Trueblood, T. Leljedal, D. Wissmiller, A. Nasta, S. Siddique, F. de la Chesnaye and D. McCollum, "LCRI Net-Zero 2050: U.S. Economy-Wide Deep Decarbonization Scenario Analysis," EPRI, Palo Alto, CA, 2022.

[6] J. H. Williams, R. Jones, B. Haley, G. Kwok, J. Hargreaves, J. Farbes and M. S. Torn, "Carbon-Neutral Pathways for the United States," *AGU Advances,* vol. 2, 2021.

[7] D. Huppman, E. Kriegler, V. Krey, K. Riahi and J. Rogelj, "IAMC 1.5C Scenario Explorer and Data hosted by IIASA," Integrated Assessment Modeling Consortium & International Institute forApplied Systems Analysis, 2018.

[8] NREL, "2022 Annual Technology Baseline," National Renewable Energy Laboratory, Golden, CO, 2022.

[9] IEA, "Global Energy and Climate Model Documentation 2023," International Energy Agency, Paris, 2023.

[10] IEA, "The Future of Hydrogen," International Energy Agency, 2019.

[11] P. O'Rourke, B. K. Mignone, P. Kyle, B. Chapman, J. Fuhrman, P. Wolfram and H. McJeon, "Supply and Demand Drivers of Global Hydrogen Deployment in the Transition toward a Decarbonized Energy System," *Environmental Science & Technology,* 2023.

[12] NREL, "H2A: Hydrogen Analysis Production Models," National Renewable Energy Laboratory, Golden, CO, 2018.

[13] E. Lewis, S. McNaul, M. Jameison, M. S. Henriksen, H. S. Matthews, J. White, L. Walsh, J. Grove, T. Shultz, T. J. Skone and R. Stevens, "Comparison of Commercial, State-of-the-art, Fossil-based Hydrogen Production Technologies," National Energy Technology Laboratory, Pittsburgh, 2022.

[14] M. Muratori, H. Kheshgi, B. Mignone, L. Clarke, H. McJeon and J. Edmonds, "Carbon capture and storage across fuels and sectors in energy system transformation pathways," *International Journal of Greenhouse Gas Control,* pp. 34-41, 2017.

[15] IEA Bioenergy, "Advanced Biofuels - Potential for Cost Reduction," International Energy Agency (IEA) Bioenergy, 2020.

[16] T. G. Kreutz, E. D. Larson, C. Elsido, E. Martelli, C. Greig and R. H. Williams, "Techno-economic prospects for producting Fischer-Tropsch jet fuel and electricity from lignite and woody biomass with CO2 capture for EOR," *Applied Energy,* vol. 279, 2020.

[17] G. Zang, P. Sun, A. A. Elgowainy, A. Bafana and M. Wang, "Performance and cost analysis of liquid fuel production from H2 and CO2 based on the Fischer-Tropsch process," *Journal of CO2 Utilization,* vol. 46, 2021.

[18] D. F. DeCarolis, P. Jaramillo, J. X. Johnson, D. L. McCollum and E. Trutnevyte, "Leveraging Open-Source Tools for Collaborative Macro-energy System Modeling Efforts," *Joule,* vol. TnQ, pp. 2523-2531, 2020.
